# Awareness of Breast Cancer and Its Risk Factors Among Medical Students of North India

**DOI:** 10.7759/cureus.103514

**Published:** 2026-02-12

**Authors:** Stacey A Marbaniang, Shreya Marcus, Pamela A Kingsley, Micheal Deodhar

**Affiliations:** 1 Surgery, Christian Medical College and Hospital, Ludhiana, IND; 2 Radiation Oncology, Christian Medical College and Hospital, Ludhiana, IND

**Keywords:** awareness, breast cancer, medical students, risk factors, screening

## Abstract

Introduction

Breast cancer remains one of the leading causes of cancer-related mortality among women in India. Early detection and prevention are crucial, and medical students play a vital role in promoting awareness.

Objective

This study assessed how well undergraduate medical students in North India understand breast cancer symptoms, risk factors, screening, and treatment.

Methods

A cross-sectional study using a 30-item, semi-structured, self-developed questionnaire was distributed through Google Forms. The questions covered general awareness; biological, reproductive, and lifestyle risk factors; as well as screening practices. Students from different MBBS academic years participated. Responses were analyzed using GraphPad Prism 8.0.2, applying descriptive statistics and chi-square tests to identify associations, with significance set at p < 0.05.

Results

A total of 307 students responded, nearly two-thirds of whom were female. The most recognized key risk factors were family history (94.1%), smoking and alcohol consumption (86.3%), and childhood radiation exposure (87.3%). Awareness of biological risks such as early menstruation (71.7%) and late menopause (67.4%) was moderate. However, knowledge about reproductive risks was weaker: only 58% recognized nulliparity, 59% were aware of hormone replacement therapy as a risk factor, and 55% identified late first childbirth. Screening knowledge showed similar gaps. While 87% had heard of breast self-examination (BSE), only one-third knew the correct timing. Less than half were aware that mammography is advised in the 40s and 50s, and only 66% realized that breast cancer can occur without pain or a lump.

Conclusion

Undergraduate medical students in North India demonstrated moderate awareness of breast cancer, with notable gaps in understanding lifestyle-related risk factors and screening methods. Strengthening medical curricula and integrating breast cancer awareness programs are essential to enhance students’ knowledge and their future role in community health promotion.

## Introduction

Among women, breast cancer is the most commonly diagnosed malignancy and remains one of the leading causes of cancer-related mortality worldwide [1]. In India, it has now surpassed cervical cancer as the most common malignancy among women. Disturbing trends indicate that it is being diagnosed at a progressively younger age, with many cases affecting women in their 30s and 40s [2, 3].

This is considered to result from a combination of lifestyle transitions, environmental influences, and inherited susceptibility [4]. The risk for breast cancer includes both non-modifiable and modifiable factors. Non-modifiable risk factors include female gender, advancing age, family history, and genetic factors such as BRCA1 and BRCA2 mutations [1,4,5]. Prolonged exposure to estrogen, secondary to early menarche, late menopause, first childbirth after 35 years of age, nulliparity, and not breastfeeding, increases the risk of breast cancer [6]. On the other hand, lifestyle-related risk factors include obesity, alcohol use, radiation exposure, and long-term use of hormonal therapies [4]. Even though systematic screening and mammography programs have significantly enhanced early detection in developed countries, the majority of Indian women present with advanced-stage disease at the time of diagnosis [7]. This often relates to a lack of awareness, social and cultural inhibitions, and limited access to screening facilities [7, 8].

Medical students are an important target group in initiatives aimed at strengthening awareness and early detection of breast cancer. Their knowledge and attitudes translate directly into patient and community education by future physicians. Previous studies from India and other low- and middle-income countries have reported incomplete knowledge of breast cancer risk factors, breast self-examination(BSE), and screening recommendations among medical students [9]. This study aimed to assess the awareness of breast cancer risk factors, symptoms, and screening practices among undergraduate medical students in a tertiary institution in North India.

## Materials and methods

This cross-sectional study was conducted among undergraduate medical students at Christian Medical College (CMC), Ludhiana, North India, to assess knowledge of breast cancer, its risk factors, and awareness of symptoms and screening practices. The 30-item semi-structured questionnaire used in this study was originally developed by the authors based on a literature review and expert consultation. It consisted of closed-ended items, including multiple-choice questions and Yes/No/Not sure response formats, designed to assess knowledge, attitudes, and behaviors toward breast cancer risk factors, symptoms, screening, and treatment. It was not adapted from any copyrighted or proprietary material. As a result, the instrument is free to use and does not require any licensing or authorization. To ensure validity and reliability, a pilot test was conducted with 20 students selected randomly across four academic years (2020-2024) and was also reviewed by faculty advisers. Students who participated in the pilot test were excluded from the final study sample, and the data were not included in the analysis. Feedback on the clarity, relevance, and comprehensibility of the questions was obtained, and necessary modifications were made. The final questionnaire covered demographic information and awareness of risk factors (genetic, reproductive, lifestyle, and hormonal), symptoms, and screening practices.

The survey was administered electronically via Google Forms to 307 medical students who met the inclusion criteria. The survey link was circulated to eligible MBBS students through institutional class-based messaging platforms. Participation was voluntary, and responses were submitted electronically. Confidentiality was maintained, and no personally identifiable information was collected. Data collection was completed over one month beginning February 25, 2025. Following the study, an educational session on breast cancer awareness and BSE was conducted by the Department of Surgery, CMC Ludhiana, as part of a health education initiative.

Ethical approval for this study was obtained from the Institutional Ethics Committee of Christian Medical College and Hospital, Ludhiana (Ref. No. IECBMHR/202502-099/Apprvl-Student-Proj/CMC & H, dated 25 February 2025). Participation was voluntary and anonymous.

All undergraduate medical students at Christian Medical College (CMC), Ludhiana, were eligible to participate in the study. Interns were excluded to ensure that participants’ academic exposure was uniform, as their additional clinical exposure, particularly in surgery and oncology, could have biased awareness levels compared with undergraduate MBBS students.

Sample size calculation

Using OpenEpi version 3, the minimum required sample size was calculated based on a previously reported awareness level of 73% among university students. At 80% power, a 95% CI, and an 8% margin of error were selected to balance statistical precision and feasibility; the required sample size was 303 [9]. To account for non-responses or incomplete submissions, 375 students were approached, of whom 307 provided complete responses and were included in the final analysis.

Statistical analysis

All collected data were first checked for completeness and consistency before analysis, and questionnaires with incomplete or missing data were excluded from the final analysis; only fully completed responses were included. Descriptive statistics, including frequencies, percentages, means, and SDs, were used to summarize demographic characteristics and responses to knowledge, awareness, and risk perception items. Categorical variables were presented as frequencies and percentages, while continuous variables were expressed as means ± SDs.

Breast cancer awareness was assessed using a 30-point scale, with one point awarded for each correct answer, zero points for each incorrect answer, and 0.25 points for each “not sure” answer. It was categorized into three levels: low (0-15; 0%-52%), moderate (16-25; 53%-85%), and high (26-30; 86%-100%). Scores ≤52% were classified as low awareness, 53%-85% as moderate awareness, and ≥86% as high awareness. These cut-offs were chosen to distinguish between limited, partial, and comprehensive knowledge in an educational assessment. Associations between sociodemographic variables and awareness levels were evaluated using inferential statistics. The chi-square test of independence was applied for relationships between categorical variables. For comparisons between two independent groups with non-normally distributed data, the Mann-Whitney U test was used. Comparisons among three or more groups were performed using the Kruskal-Wallis test, followed by Dunn’s multiple comparisons post hoc test with Bonferroni correction. Normally distributed continuous variables across three or more groups were compared using one-way ANOVA. A p-value of <0.05 was considered statistically significant. All statistical analyses were performed using GraphPad Prism version 8.0.2 (GraphPad Software, San Diego, CA, USA).

## Results

Sociodemographic characteristics of participants

A total of 307 medical students participated in the study. As shown in Table 1, most participants were female (199, 64.8%), while males constituted 108 (35.2%). The highest representation was from the 2022 batch (third professional year; 99, 32.3%), followed by the 2024 batch (first professional year; 89, 29.0%) and the 2023 batch (second professional year; 69, 22.5%). The 2020 and 2021 batches had fewer respondents (fourth professional year and third part two professional year: 11, 3.6%, and 39, 12.7%, respectively).

**Table 1:** Sociodemographic characteristics of participants (N = 307).

Variable	Categories	Frequency (N)	Percentage (%)	Statistical test used
Gender	Male	108	35.2	Descriptive (frequency, %)
	Female	199	64.8	
	Total	307	100	
MBBS batch	2020	11	3.6	Descriptive (frequency, %)
	2021	39	12.7	
	2022	99	32.3	
	2023	69	22.5	
	2024	89	29	
	Total	307	100	

Gender distribution across MBBS batches

As shown in Table 2, the gender distribution across MBBS batches from 2020 to 2024 showed some differences. In the 2020 batch, females made up 72.73% of the students, while males accounted for 27.27%. This gap widened further in the 2021 batch, where almost all students were female (92.31%) and only 7.69% were male. The 2022 batch was more evenly balanced, with 41.41% males and 58.59% females, and a similar pattern was seen in the 2024 batch, with 41.57% males and 58.43% females. The 2023 batch also showed a female majority, comprising 65.22% females and 34.78% males. Overall, females consistently outnumbered males across all batches. However, although these differences appeared evident, the chi-square test (χ² = 16.51, df = 10, p = 0.0859) indicated that the variation in gender distribution across batches was not statistically significant.

**Table 2:** Gender distribution across MBBS batches (2020-2024), showing the number (N) and percentage of males and females within each batch. A chi-square test was performed to assess the association between gender and batch. A p-value < 0.05 was considered statistically significant.

Variable	Categories	Male (N)	Female (N)	Total (N)	% Male (within batch)	% Female (within batch)	Statistical test used
MBBS batch	2020	3	8	11	27.3	72.7	Chi-square statistic (χ²): 16.51, df: 10, p-value = 0.0859
	2021	3	36	39	7.7	92.3	
	2022	41	58	99	41.4	58.6	
	2023	24	45	69	34.8	65.2	
	2024	37	52	89	41.6	58.4	

Awareness of breast cancer risk factors

Family and Personal History-Related Risk Factors

As shown in Table 3, awareness was highest for a family history of breast cancer, with 94.14% of participants responding “Yes,” while only 3.26% said “No” and 2.61% were unsure. Awareness was moderate regarding benign breast disease, where 73.94% correctly identified it as a risk factor, 10.75% did not, and 15.31% were uncertain. For long-term use of oral contraceptives, 67.10% recognized it as a risk factor, 7.82% disagreed, and 25.08% were unsure. The lowest awareness was observed for postmenopausal hormone therapy, with only 58.96% identifying it as a risk factor, 9.45% denying any risk, and a relatively high 31.60% expressing uncertainty.

**Table 3:** Awareness of family and personal history-related risk factors for breast cancer. Awareness levels were classified based on the total score out of 30 as high awareness (score 26–30; 86%–100%), moderate awareness (score 16-25; 53%-85%), and low awareness (score 0-15; 0%-52%).

SI No.	Question	Yes (%)	No (%)	Not sure (%)	Interpretation
1	Do you think a history of breast cancer in the family can pose as a risk factor for breast cancer?	289 (94.14%)	9 (3.26%)	9 (2.61%)	High awareness about familial risk.
2	Do you think a history of benign breast disease can increase the chances of having breast cancer?	227 (73.94%)	33 (10.75%)	47 (15.31%)	Moderate awareness; notable uncertainty.
3	Do you think using oral contraceptives for more than 5 years increases the chances of having breast cancer?	206 (67.10%)	24 (7.82%)	77 (25.08%)	Fair awareness, but one-fourth unsure.
4	Do you think hormone therapy after menopause may lead to breast cancer?	181 (58.96%)	29 (9.45%)	97 (31.60%)	Fair awareness and high uncertainty.

Lifestyle and Environmental Risk Factors

As shown in Table 4, participants demonstrated varying levels of awareness regarding lifestyle and environmental factors linked to breast cancer. Most respondents (n = 268; 87.30%) correctly identified radiation exposure to the chest or breast during childhood or adolescence as a risk factor, and a similar proportion (86.32%) recognized smoking and alcohol consumption as potential risks, reflecting high awareness in these areas. However, awareness dropped sharply for low physical activity, with only 34.53% agreeing it could increase breast cancer risk, while 34.85% disagreed and 30.62% were unsure, indicating uncertainty about the protective benefits of exercise. Awareness of obesity or being overweight was moderate, with 52.12% agreeing, though 25.08% disagreed and 22.80% were uncertain. Similarly, awareness about stress and night shift work (51.79%) and dietary factors such as red meat and fatty food intake (51.47%) was borderline moderate, with a considerable proportion of participants uncertain (26.38% and 33.55%, respectively).

**Table 4:** Awareness of lifestyle and environmental risk factors for breast cancer. Awareness levels were classified based on the total score out of 30 as high awareness (score 26-30; 86%-100%), moderate awareness (score 16-25; 53%-85%), and low awareness (score 0-15; 0%-52%).

SI No.	Question	Yes (%)	No (%)	Not sure (%)	Interpretation
5	Do you think exposure to radiation concerning the chest or breast during childhood or adolescence can lead to breast cancer?	268 (87.30%)	12 (3.91%)	27 (8.79%)	High awareness of radiation risk.
6	Do you think low physical activity can lead to breast cancer?	106 (34.53%)	107 (34.85%)	94 (30.62%)	Very low awareness; responses almost evenly distributed.
7	Do you think obesity or being overweight may lead to breast cancer?	160 (52.12%)	77 (25.08%)	70 (22.80%)	Moderate awareness, with a notable proportion uncertain.
8	Do you think smoking and consumption of alcohol may lead to breast cancer?	265 (86.32%)	17 (5.54%)	25 (8.14%)	High awareness of behavioral risks.
9	Do you think stress and working night shifts can be factors that can cause breast cancer?	159 (51.79%)	67 (21.82%)	81 (26.38%)	Low awareness, with over a quarter unsure.
10	Do you think high consumption of red meat and fatty foods contributes to the increasing incidence of breast cancer?	158 (51.47%)	46 (14.98%)	103 (33.55%)	Low awareness; one-third unsure.

Biological and Reproductive Risk Factors

As shown in Table 5, participants demonstrated varying levels of awareness regarding biological and reproductive risk factors for breast cancer. A large majority (81.11%) correctly recognized that the risk of breast cancer increases with age, indicating high awareness in this area. However, awareness was considerably lower for reproductive factors. Only 50.81% acknowledged that lack of breastfeeding increases breast cancer risk, while 24.10% disagreed and 25.08% were unsure. Similarly, 50.16% identified early menarche or late menopause as risk factors, but nearly one-fourth (26.71%) were uncertain. Awareness was lowest for late first childbirth, with just 41.37% agreeing it increases risk, while 25.08% disagreed and 33.55% were unsure.

**Table 5:** Awareness of biological and reproductive risk factors for breast cancer. Awareness levels were classified based on the total score out of 30 as high awareness (score 26-30; 86%-100%), moderate awareness (score 16-25; 53%-85%), and low awareness (score 0-15; 0%-52%).

SI No.	Question	Yes (%)	No (%)	Not sure (%)	Interpretation
11	Do you think the risk of breast cancer increases with age?	249 (81.11%)	25 (8.14%)	33 (10.75%)	High awareness of age-related risk.
12	Do you think lack of breastfeeding increases the incidence of having breast cancer?	156 (50.81%)	74 (24.10%)	77 (25.08%)	Low awareness with significant uncertainty.
13	Do you think experiencing early menarche (<12 years) or late menopause (>55 years) increases the risk of developing breast cancer?	154 (50.16%)	71 (23.13%)	82 (26.71%)	Low awareness; nearly one-fourth unsure.
14	Do you believe that having a first child at an older age (>35 years) increases the risk of developing breast cancer?	127 (41.37%)	77 (25.08%)	103 (33.55%)	Low awareness with high uncertainty.

Awareness of symptoms of breast cancer

As shown in Table 6, awareness of breast cancer symptoms varied among participants. Most respondents (83.71%) correctly identified a painless, palpable breast lump as a warning sign. Awareness was fair for other symptoms such as a lump under the armpit (66.78%), nipple discharge (71.99%), and nipple retraction (72.64%), although about one in five participants were unsure. Fewer participants recognized skin-related changes, with only 62.21% identifying nipple discoloration and 57.00% recognizing redness of the breast as possible signs.

**Table 6:** Awareness of common signs and symptoms of breast cancer. Awareness levels were classified based on the total score out of 30 as high awareness (score 26-30; 86%-100%), moderate awareness (score 16-25; 53%-85%), and low awareness (score 0-15; 0%-52%).

SI No.	Question	Yes (%)	No (%)	Not sure (%)	Interpretation
15	Do you think a painless and palpable breast lump may indicate breast cancer?	257 (83.71%)	25 (8.14%)	25 (8.14%)	High awareness of a key warning sign.
16	Do you think a painless mass under the armpit indicates breast cancer?	205 (66.78%)	42 (13.68%)	60 (19.54%)	Moderate awareness with some uncertainty.
17	Is bleeding or discharge from the nipple a sign of breast cancer?	221 (71.99%)	18 (5.86%)	68 (22.15%)	Fair awareness but notable uncertainty.
18	Does unilateral new-onset retraction of the nipple indicate breast cancer?	223 (72.64%)	18 (5.86%)	66 (21.50%)	Fair awareness with room for improvement.
19	Is discoloration around the nipple a sign of breast cancer?	191 (62.21%)	31 (10.10%)	85 (27.69%)	Moderate awareness; over a quarter uncertain.
20	Can breast cancer present with redness of the breast skin?	175 (57.00%)	33 (10.75%)	99 (32.25%)	Low to moderate awareness; high uncertainty.
21	Do you think abrupt changes in the size and shape of the breast indicate breast cancer?	206 (67.10%)	34 (11.07%)	67 (21.82%)	Moderate awareness; one-fifth unsure.

Screening awareness of breast cancer

As shown in Table 7, nearly all participants (97.72%) recognized the importance of screening in the early detection of breast cancer. However, only 37.79% believed that BSE alone is sufficient for screening, showing moderate understanding of comprehensive screening methods. Although 76.22% correctly identified “after 20 years” as the right time to start BSE, only 30.29% knew it should be done “one week after menstruation,” while more than half (54.72%) were unsure. Furthermore, 56.68% reported that BSE should be performed monthly, yet self-practice remained low (27.04%). Awareness regarding mammography was also limited, as only 28.66% selected “after 30 years,” while 45.28% thought it should be done after 40 years. Although most participants had heard of BSE, fewer students were aware of the correct technique, which is systematic inspection and palpation using the finger pad, and the recommended timing of monthly examination, ideally 5-7 days after menstruation, highlighting an important gap between awareness and practical knowledge.

**Table 7:** Awareness related to breast cancer screening and self-examination. Awareness levels were classified based on the total score out of 30 as high awareness (score 26-30; 86%-100%), moderate awareness (score 16-25; 53%-85%), and low awareness (score 0-15; 0%-52%).

SI No.	Question	Yes (%)	No (%)	Not sure (%)	Interpretation
22	Do you think screening helps in the early detection of breast cancer?	300 (97.72%)	1 (0.33%)	6 (1.95%)	Very high awareness of the role of screening.
23	Do you think breast self-examination is sufficient as a screening method for breast cancer?	116 (37.79%)	164 (53.42%)	27 (8.79%)	Moderate understanding that BSE alone is insufficient.
24	Do other women in your family perform breast self-examinations?	83 (27.04%)	123 (40.07%)	101 (32.90%)	Low practice of BSE in families; high uncertainty.
25	When is the best time to start breast self-examination?	After 20 years: 234 (76.22%)	After 40 years: 31 (10.10%)	42 (13.68%)	Moderate awareness of when to initiate BSE.
26	When is the best time to do a breast self-examination during the menstrual cycle?	One week after menstruation: 93 (30.29%)	One week before menstruation: 46 (14.98%)	168 (54.72%)	Poor awareness; over half uncertain.
27	How often should a breast self-examination be done?	Monthly: 174 (56.68%)	Quarterly: 69 (22.48%)	64 (20.85%)	Moderate awareness of recommended frequency.
28	When is the best time for mammography?	After 30 years: 88 (28.66%)	After 40 years: 139 (45.28%)	80 (26.06%)	Low awareness regarding mammography timing.

Treatment awareness of breast cancer

As shown in Table 8, most participants demonstrated good awareness of evidence-based treatment for breast cancer. A large majority (78.83%) correctly recognized that combining surgery with chemotherapy improves survival, reflecting a clear understanding of the benefits of multimodal therapy. However, only 9.77% believed that homeopathy or Ayurveda are preferred treatments, while 60.91% disagreed, showing confidence in scientifically validated medical care. Still, some respondents (18.24% and 29.32%, respectively) were unsure, indicating lingering misconceptions and a need for stronger education on appropriate treatment approaches.

**Table 8:** Awareness regarding breast cancer treatment approaches. Awareness levels were classified based on the total score out of 30 as high awareness (score 26-30; 86%-100%), moderate awareness (score 16-25; 53%-85%), and low awareness (score 0-15; 0%-52%).

SI No.	Question	Yes (%)	No (%)	Not sure (%)	Interpretation
29	Is combining surgery with chemotherapy leading to improvement of survival in breast cancer?	242 (78.83%)	9 (2.93%)	56 (18.24%)	High awareness of the effectiveness of combined cancer treatments.
30	Do you think homeopathy and ayurveda are preferred for treating breast cancer?	30 (9.77%)	187 (60.91%)	90 (29.32%)	Most respondents favor evidence-based treatment; confusion remains.

Gender-based comparison of breast cancer awareness responses and scores

**Figure 1:**
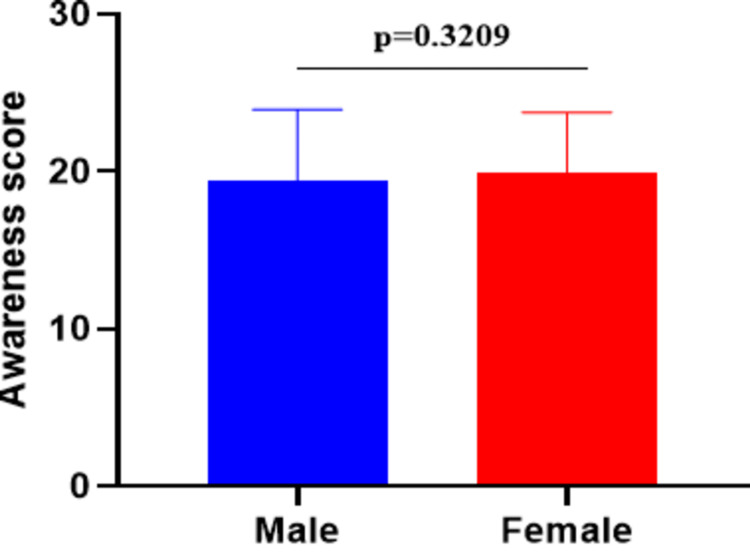
Comparison of breast cancer awareness scores between male and female participants using the Mann-Whitney U test. Data are presented as mean ± SD. A p-value < 0.05 was considered statistically significant.

As shown in Figure 1, the Mann-Whitney U test was conducted to compare overall breast cancer awareness scores between male and female participants. The results indicated no statistically significant difference between the two groups (p = 0.3209), suggesting comparable awareness levels. Additionally, a chi-square test of independence was performed to examine the association between gender and responses to the 30 individual awareness questions shown in Table 9. Among these, only Question 24 (“Do other women in your family perform breast self-examination?”) showed a statistically significant association with gender (χ² = 15.44, p = 0.0171). All other questions (Q1-Q23 and Q25-Q30) showed no significant gender-based differences (p > 0.05), indicating a generally consistent response pattern across both genders.

**Table 9:** Gender-based comparison of breast cancer awareness responses. A p-value < 0.05 was considered statistically significant. BSE: Breast self-examination.

Risk factor	Yes (%)	No (%)	Not sure (%)	χ²	df	p-value
Family history	73.9	3.2	2.6	1.934	6	0.3801
Benign breast disease		10.8	15.3	2.767	6	0.8375
Oral contraceptive use (>5 years)	67.1	7.8	25.1	1.308	6	0.9712
Postmenopausal hormone therapy	59	9.4	31.6	6.749	6	0.3447
Radiation exposure	87.3	4	8.8	3.669	6	0.7214
Smoking and alcohol	86.3	5.5	8.1	6.371	6	0.3829
Low physical activity	34.5	34.8	30.6	0.0388	6	>0.9999
Obesity	52.1	25.1	22.8	6.528	6	0.3667
Stress/night shift	52	21.8	26.3	1.168	6	0.9784
Consumption of red meat and fatty foods	51.5	15	33.5	0.7862	6	0.9924
Increasing age	81.1	8.1	10.7	3.633	6	0.7261
Lack of breastfeeding	50.8	24.1	25.1	6.695	6	0.35
Early menarche/late menopause	50.2	23.1	26.7	4.43	6	0.6187
Late first childbirth	41.4	25.1	33.5	3.857	6	0.696
Painless and palpable breast lump	83.7	8.1	8.1	0.2961	6	0.9995
Axillary painless mass	66.8	13.7	19.5	2.482	6	0.8705
Nipple discharge or bleeding	72	5.8	22.1	4.791	6	0.5708
Unilateral new-onset nipple retraction	72.6	5.8	21.5	5.933	6	0.4308
Nipple/areola discoloration	62.2	10.1	27.7	3.626	6	0.7271
Skin redness	57	10.7	32.2	0.4098	6	0.9988
Abrupt change in size and shape of breast	67.1	11.1	21.8	0.2114	6	0.9998
Importance of screening	97.7	0.3	1.9	0.5548	6	0.9971
BSE sufficiency	37.8	53.4	8.8	2.184	6	0.902
Family BSE practice	27	40.1	32.9	15.44	6	0.0171
Start BSE	76.2	10.1	13.7	0.9401	6	0.9878
Correct timing for BSE (post-menstrual week)	30.3	15	54.7	0.2112	6	0.9998
BSE frequency	56.7	22.5	20.8	1.163	6	0.9787
Best time for mammography	28.7	45.3	26	2.099	6	0.9104
Surgery and chemotherapy improves survival	78.8	2.9	18.3	1.56	6	0.9554
Homeopathy/Ayurveda preferred	9.8	60.9	29.3	4.11	6	0.6618

Breast cancer awareness across MBBS batches

Variation in Breast Cancer Awareness Scores Among MBBS Batches

**Figure 2:**
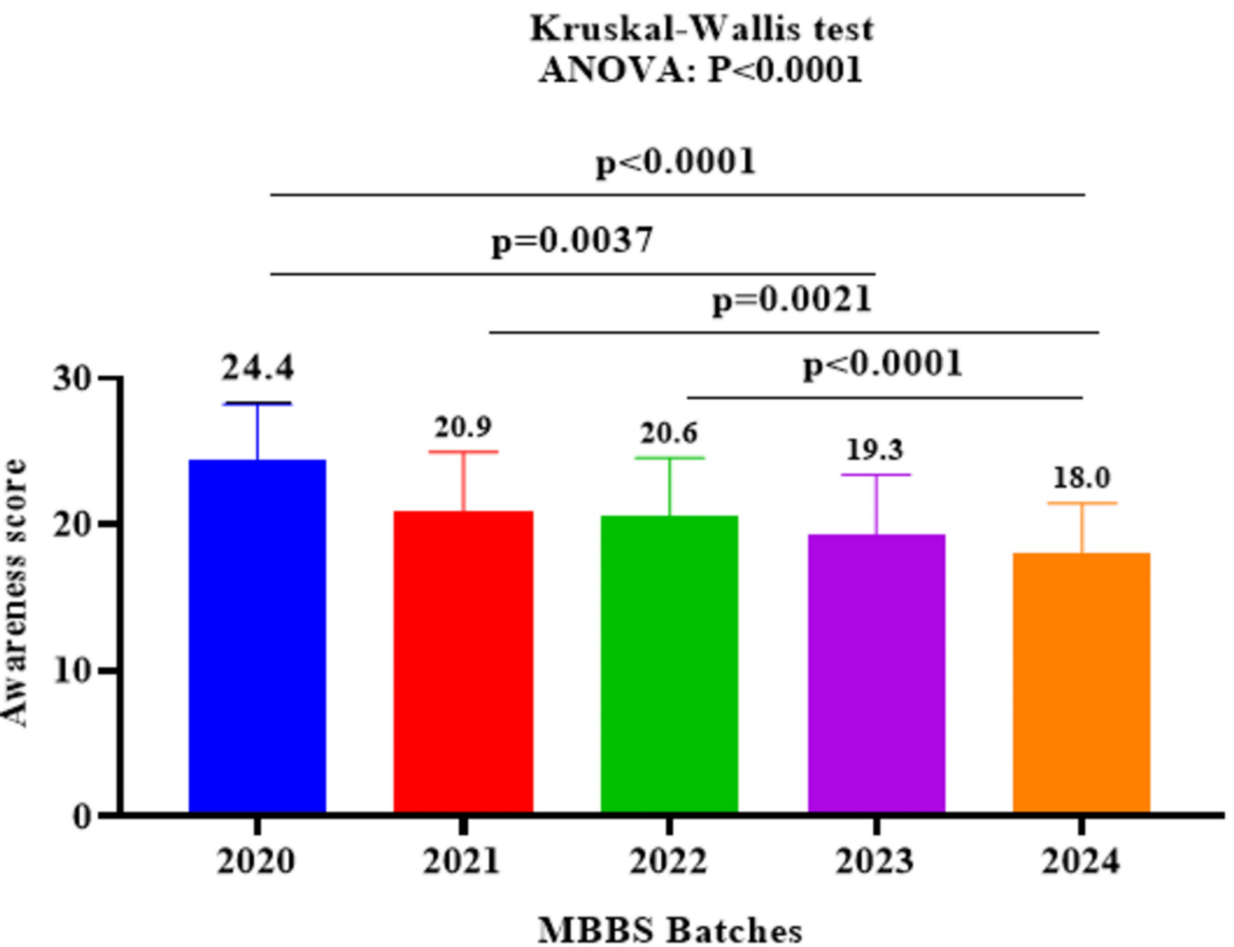
Variation in breast cancer awareness scores among MBBS batches. Data are presented as mean ± SD. A p-value < 0.05 was considered statistically significant.

Figure 2 presents the comparison of breast cancer awareness scores across the five MBBS batches using the Kruskal-Wallis test, which revealed a statistically significant difference in awareness levels among the groups (p < 0.0001). This indicates that breast cancer awareness was not uniformly distributed across batches. Post hoc analysis using Dunn’s multiple comparisons test further pinpointed where these differences lay. The 2024 batch demonstrated significantly lower awareness scores compared to the 2020 (p < 0.0001), 2021 (p = 0.0021), and 2022 (p < 0.0001) batches. Additionally, the 2023 batch scored significantly lower than the 2020 batch (p = 0.0037). No significant differences were observed among the 2020, 2021, and 2022 batches, nor between the 2023 and 2024 batches. These findings suggest that earlier batches, particularly those from 2020 to 2022, possessed higher levels of breast cancer awareness compared to more recent cohorts, with the 2024 batch showing the lowest overall scores.

Chi-Square Analysis of Breast Cancer Awareness Responses Across MBBS Batches

Table 10 explores batch-wise response patterns to individual questions using chi-square analysis. Significant differences in responses were found for Questions 4, 9, 10, 11, and 29 (p < 0.05), indicating variable awareness or understanding among batches on these specific topics. The most notable inter-batch disparities were seen in Q4, Q9, and Q10, where the p-values were extremely low (≤ 0.0001), reflecting highly significant variability. In contrast, responses to the remaining questions (Q1-Q3, Q5-Q8, Q12-Q23, Q24-Q28, and Q30) did not differ significantly among batches (p ≥ 0.05), suggesting a relatively consistent level of awareness across cohorts for those items.

**Table 10:** Chi-square analysis of breast cancer awareness questions across MBBS batches.

SI No.	Question No.	χ² value	Degrees of freedom (df)	p-value	Significance
1	Q4	43.25	15	0.0001	Significant (p < 0.05)
2	Q9	58.86	15	<0.0001	Significant (p < 0.05)
3	Q10	49.4	15	<0.0001	Significant (p < 0.05)
4	Q11	26.25	15	0.0354	Significant (p < 0.05)
5	Q29	26.97	15	0.029	Significant (p < 0.05)
6	Q1-Q3, Q5-Q8, Q12-Q23, Q24-Q28, Q30	2.877-23.64	15	0.0715-0.9997	Not significant (p ≥ 0.05)

To identify which batch pairs contributed to the observed differences, Table 11 details post hoc pairwise comparisons. For Questions 4, 9, 10, and 11, consistent and statistically significant differences were observed between Batch 2020 and Batch 2024, with p-values ranging from 0.0002 to 0.0009. These results point to a marked decline in awareness or confidence among students in the most recent batch. In the case of Question 29, a significant difference emerged between Batches 2022 and 2023 (p = 0.0148), suggesting a fluctuation in knowledge or behavior between these adjacent years. Overall, these pairwise differences underscore the importance of reinforcing breast cancer education, especially for more recent cohorts such as the 2024 batch.

**Table 11:** Post hoc pairwise comparisons of awareness responses between MBBS batches.

SI No.	Question	Batch pair	χ² value	df	p-value	Statistically significant?	Interpretation
1	Q4	Batch 2020 vs Batch 2024	14.47	2	0.0007	Yes	A highly significant difference in responses; Batch 2024 had notably more “Not sure” responses.
2	Q9	Batch 2020 vs Batch 2024	15.81	2	0.0004	Yes	A strong difference was observed; Batch 2024 likely shows less awareness or more uncertainty in responses.
3	Q10	Batch 2020 vs Batch 2024	17.25	2	0.0002	Yes	Marked difference in understanding/awareness between the earliest and most recent batch.
4	Q11	Batch 2020 vs Batch 2024	14.1	2	0.0009	Yes	Batch 2024 significantly differs from Batch 2020; there were less confident or informed responses.
5	Q29	Batch 2022 vs Batch 2023	8.429	2	0.0148	Yes	Indicates differences in awareness or behavior related to Q29 between these two mid-level batches.

## Discussion

Summary of the key findings

Knowledge of breast cancer risk factors, symptoms, screening, and treatment among medical students in India was moderate overall. However, there were several significant gaps in students’ understanding of some reproductive and hormonal factors, lifestyle factors, and evidence-based screening practices. The increase in awareness of breast cancer across academic years indicates that continued clinical training is associated with greater awareness of breast cancer.

Interpretation

Medical students have limited awareness of cancer-related topics, despite their training. They have good knowledge of genetics and family history. However, medical students do not appear to retain modifiable lifestyle factors or reproductive risk factors associated with cancer. This gap may be due to the relative newness and increasing relevance of modifiable risk factors associated with the growing cancer burden in India. There is also a gap between theoretical education and practical training when it comes to BSE, including timing, technique, and screening recommendations. Future physicians play a crucial role in educating communities and raising awareness about cancer in resource-constrained settings. However, gaps in their knowledge may limit their ability to promote early detection and reduce cancer risk in their communities.

Comparison with existing study

This study included a total of 307 participants; nearly two-thirds were female (64.8%). This is comparable to Yadav P and Jaroli DP (2020) [6], in which a predominance of female participants was observed in their study on breast cancer awareness among college-going women. Akter MF and Ullah MO (2022) [9] also observed a female-majority cohort in Bangladesh, suggesting a consistent trend across South Asia. Gender distribution varied across batches, with female dominance in some cohorts and a more balanced ratio in others. However, the chi-square test showed no statistically significant difference in gender distribution across years. This is comparable to the findings of Akter MF and Ullah MO (2022) [9], who noted that gender distribution did not significantly affect overall awareness scores among students. A majority of respondents correctly identified family history as a key risk factor. This is consistent with Yadav P and Jaroli DP (2020) [6] and Akter MF and Ullah MO (2022) [9], in which high awareness of genetic predisposition among young women and medical students was observed. Students demonstrated stronger awareness of risk factors such as radiation exposure during adolescence and tobacco/alcohol use. This aligns with results reported by Yadav P and Jaroli DP (2020) [6], where 84% recognized tobacco and alcohol as risks, and Akter MF and Ullah MO (2022) [9], where over 80% of students recognized radiation exposure.

Students demonstrated recognition of classical symptoms, identifying a painless breast lump, followed by nipple retraction and nipple discharge. However, awareness of less typical signs, such as skin redness and nipple discoloration, was weaker, with many students reporting uncertainty. In a similar study by Pradhan A et al. (2017) [10], it was emphasized that delayed recognition of symptoms, especially atypical symptoms, results in presentation at advanced stages and leads to a poor prognosis. Similarly, Koo MM et al. (2017) [11] highlighted that atypical symptoms such as skin changes or nipple abnormalities were frequently missed, leading to longer diagnostic intervals. Nearly all respondents agreed that screening is essential for early detection, reflecting positive attitudes toward preventive care. However, misconceptions were evident. While the majority reported that BSE should begin after age 20, only some identified the correct timing, one week after menstruation, with more than half uncertain. Similarly, only 28.7% knew the correct age for initiating mammography (40 years). This is comparable to Akter MF and Ullah MO (2022) [9], where knowledge of proper BSE technique and mammography guidelines was limited. Hendrick RE and Monticciolo DL (2024) [12] emphasized that mammography should begin at 40 years, reinforcing the importance of aligning medical student knowledge with current guidelines. Kassie AM et al. (2021) [13] further confirmed that while awareness of BSE was high among female university students in Ethiopia, actual practice remained low.

Most students correctly recognized surgery combined with chemotherapy as the most effective treatment, reflecting acceptance of multimodal evidence-based management. Encouragingly, only a small proportion reported homeopathy or Ayurveda as preferred options, and the majority rejected them. This is comparable to Pradhan A et al. (2017) [10], who emphasized the importance of multimodal therapy in improving survival outcomes. Some participants were uncertain about alternative therapies, suggesting susceptibility to misinformation. A similar concern was noted by Johnson SB and Bylund CL (2023) [14], who warned of the persistence of misinformation in cancer care. Strengthening clinical skills and knowledge may help consolidate critical thinking and evidence-based decision-making among medical students.

Strengths

This research has several strengths, including the inclusion of medical students from various years of training, allowing analysis of trends and changes throughout medical education. Additionally, the study used a structured and validated questionnaire that examined multiple aspects of breast cancer awareness, enhancing the reliability of the results. Finally, due to the sufficiently large sample size and systematic analysis of the collected data, the researchers were able to draw reliable conclusions.

Limitations

This study has certain limitations. First, as a single-center, cross-sectional study, the findings may not be generalizable to all medical students across India. Second, data were collected using a self-reported questionnaire, which may be subject to recall bias and social desirability bias, potentially leading to overestimation of awareness levels. Third, although the questionnaire was validated and piloted, it primarily assessed knowledge and awareness and did not objectively evaluate actual practice or skill competency, particularly for BSE. Finally, the uneven representation of MBBS batches, especially lower participation from senior batches, may have influenced comparisons across academic years.

Future direction

Future research should include multi-center studies involving medical colleges from different regions to improve generalizability. Longitudinal studies evaluating the impact of structured educational interventions, such as workshops and hands-on BSE training, would help assess improvement in both knowledge and practice over time. Incorporating objective skill assessments and follow-up evaluations can bridge the gap between awareness and behavior. Additionally, integrating breast cancer awareness modules earlier in the MBBS curriculum and encouraging student-led community outreach programs may strengthen preventive health education and contribute to improved early detection practices in the wider population. This study emphasizes the need for structured breast cancer education modules in undergraduate medical programs. These modules should cover risk reduction, appropriate screening, and practical skills for BSE. Students’ focus on prevention can be further strengthened by opportunities to participate in community outreach and supervised clinical education. To further build on the current research, future investigations should be conducted in multi-institutional settings, including longitudinal and interventional designs, to evaluate the effectiveness of targeted breast cancer advocacy and educational initiatives. The results of such investigations can ultimately contribute to preparing medical students as proficient advocates for the prevention and early detection of breast cancer.

## Conclusions

This study demonstrates that medical students possess a good understanding of key breast cancer risk factors, particularly family history and radiation exposure. However, important knowledge gaps remain regarding the role of hormones, reproductive factors, and lifestyle influences. While awareness of common symptoms is high, recognition of less typical warning signs is limited. Although most students acknowledge the importance of screening, their knowledge of BSE techniques and mammography guidelines is insufficient. Awareness levels did not differ significantly by gender, though female students reported greater engagement in breast health practices within their families. Differences across batches suggest that clinical exposure and training play a critical role in strengthening breast cancer knowledge, with senior students demonstrating higher awareness than their junior counterparts. These findings underscore the need to enhance breast cancer education within medical curricula, particularly by emphasizing hormonal and reproductive risk factors as well as evidence-based screening practices. Strengthening medical students’ knowledge will improve their ability to educate patients, promote early detection, and ultimately contribute to better breast cancer outcomes. The impact of post-survey educational interventions on long-term awareness and screening practices was not assessed. Future studies should consider longitudinal designs with follow-up assessments to evaluate the effectiveness of breast cancer awareness programs.

## Appendices

Appendix 1

**Table 12:** Scoring table for assessing knowledge of breast cancer risk factors, signs and symptoms, screening, and treatment.

Question number	Question	Yes	No	Not sure
Section A: Risk factors of breast cancer				
Q1	Do you think a history of breast cancer in the family can pose as a risk factor for breast cancer?	1	0	0.25
Q2	Do you think a history of benign breast disease can increase the chances of having breast cancer?	1	0	0.25
Q3	Do you think using oral contraceptives for more than 5 years increases the chances of having breast cancer?	1	0	0.25
Q4	Do you think hormone therapy after menopause may lead to breast cancer?	1	0	0.25
Q5	Do you think exposure to radiation to the chest or breast during childhood or adolescence can lead to breast cancer?	1	0	0.25
Q6	Do you think low physical activity can lead to breast cancer?	1	0	0.25
Q7	Do you think obesity or being overweight may lead to breast cancer?	1	0	0.25
Q8	Do you think the risk of breast cancer increases with age?	1	0	0.25
Q9	Do you think lack of breastfeeding increases the incidence of breast cancer?	1	0	0.25
Q10	Do you think experiencing early menarche (<12 years) or late menopause (>55 years) increases the risk of developing breast cancer?	1	0	0.25
Q11	Do you believe that having a first child at an older age (>35 years) increases the risk of developing breast cancer?	1	0	0.25
Q12	Do you think smoking and consumption of alcohol may lead to breast cancer?	1	0	0.25
Q13	Do you think stress and working night shifts can be factors that can cause breast cancer?	1	0	0.25
Q14	Do you think high consumption of red meat and fatty foods contributes to the increasing incidence of breast cancer?	1	0	0.25
Section B: Signs and symptoms				
Q15	Do you think a painless and palpable breast lump may indicate breast cancer?	1	0	0.25
Q16	Do you think a painless mass under the armpit may indicate breast cancer?	1	0	0.25
Q17	Is bleeding or discharge from the nipple a sign of breast cancer?	1	0	0.25
Q18	Does unilateral new-onset retraction of the nipple indicate breast cancer?	1	0	0.25
Q19	Is discoloration around the nipple a sign of breast cancer?	1	0	0.25
Q20	Can breast cancer present with redness of the breast skin?	1	0	0.25
Q21	Do you think abrupt changes in the size and shape of the breast indicate breast cancer?	1	0	0.25
Section C: Breast screening and treatment				
Q22	Do you think screening helps in the early detection of breast cancer?	1	0	0.25
Q23	Do you think breast self-examination is sufficient as a screening method for breast cancer?	0	1	0.25
Q24	Do other women in your family perform breast self-examinations?	Practice item	Practice item	Practice item
Q25	When is the best time to start breast self-examination?	1 (After 20 years)	0 (After 40 years)	0.25
Q26	When is the best time to do a breast self-examination during the menstrual cycle?	1 (One week after menstruation)	0 (One week before menstruation)	0.25
Q27	How often should a breast self-examination be done?	1 (Monthly)	0 (Quarterly)	0.25
Q28	When is the best time for mammography?	0 (After 30 years)	1 (After 40 years)	0.25
Q29	Is combining surgery with chemotherapy leading to improved survival in breast cancer?	1	0	0.25
Q30	Do you think homeopathy and Ayurveda are preferred for treating breast cancer?	0	1	0.25
Total score: 30				
Low awareness: 0-15; Moderate awareness: 16-25; High awareness: 26-30.				
